# Comparing Artificial Intelligence and Obstetrics Residents in Answering Standardized Patient Questions Regarding Gestational Diabetes

**DOI:** 10.7759/cureus.94662

**Published:** 2025-10-15

**Authors:** Azam Faraji, Hossein Faramarzi, Mahsa Razeghi, Nasrin Asadi, Homeira Vafaei, Maryam Kasraeian

**Affiliations:** 1 Department of Obstetrics and Gynecology, Shiraz University of Medical Sciences, Shiraz, IRN; 2 Department of Community Medicine, Shiraz University of Medical Sciences, Shiraz, IRN

**Keywords:** artificial intelligence, chatbots, gestational diabetes mellitus, pregnancy, residents

## Abstract

Introduction

This study evaluated the performance of three artificial intelligence (AI) chatbots (GPT-3.5 (OpenAI, San Francisco, USA), GPT-4o (OpenAI, San Francisco, USA), and DeepSeek V3 0324 (DeepSeek AI, Beijing, China)) compared to eight gynecology residents in answering questions related to gestational diabetes mellitus (GDM), aiming to assess and compare the accuracy and completeness of responses to standardized patient questions on gestational diabetes in pregnancy.

Methods

Twenty-four questions were answered by three chatbots (GPT-3.5, GPT-4o, and DeepSeek V3 0324) and eight residents. Two faculty members independently rated the responses for accuracy and completeness using a 5-point scale. Independent-samples t-tests were used for statistical analysis.

Results

The mean accuracy scores were 3.64 for residents, 4.67 for GPT-3.5, 4.69 for GPT-4o, and 4.81 for DeepSeek V3 0324. The mean completeness scores were 2.05 for residents, 2.83 for GPT-3.5, 4.00 for GPT-4o, and 4.75 for DeepSeek V3 0324. T-tests showed that all AI models had significantly higher accuracy than residents (p < 0.001). Completeness scores were significantly higher for GPT-4o and DeepSeek V3 0324 (p < 0.001), while the difference between GPT-3.5 and residents for completeness was not statistically significant (p = 0.058).

Conclusion

AI models, particularly DeepSeek V3 0324 and GPT-4o, outperformed gynecology residents in both accuracy and completeness when answering GDM-related questions. These preliminary findings suggest that AI tools may complement medical education and clinical support, but further research is required before broader implementation.

## Introduction

Artificial intelligence (AI) in healthcare systems has significantly increased over the last few decades. Statistics indicate that investments in AI for healthcare are expected to grow eighteenfold by 2025. However, to successfully implement AI in clinical settings, collaboration among stakeholders and appropriate training will be essential [1]. In recent years, large AI models known as generative pretrained transformers (GPTs) have demonstrated impressive capabilities beyond basic text generation, including answering clinical questions and engaging in conversation. ChatGPT, developed by OpenAI, is one of the most widely recognized examples [2].

ChatGPT has had a rapid and expanding influence on the field of medicine, as seen in the sharp rise in related publications since its initial release. Much of the current research focuses on its applications in medical studies, evaluation of its accuracy, and its potential to support patient communication [3]. Notably, ChatGPT has been observed to offer responses to patient questions that are often more detailed and empathetic than those provided by experienced physicians. In a study involving 195 patient inquiries sourced from an online health forum, reviewers consistently preferred ChatGPT’s replies over those from physicians. The responses generated by ChatGPT were rated higher for both clarity and empathy, highlighting the growing potential of artificial intelligence in enhancing communication within clinical care [4]. Alongside its potential to support patients, ChatGPT may also assist physicians in care coordination and the development of treatment strategies. Thanks to its ability to rapidly synthesize information and offer contextually appropriate suggestions, it could serve as a practical tool for improving decision-making and easing the burden of routine clinical tasks [5].

AI has also shown potential in addressing clinical questions raised by healthcare professionals. In a study involving 33 physicians from 17 medical specialties, a total of 284 questions were submitted. ChatGPT provided answers that were, in many cases, both accurate and thorough. A notable share of these responses was judged to be fully correct and clinically reliable [6].

Although the adoption of AI in obstetrics and gynecology has advanced more gradually than in other medical fields, recent progress suggests promising opportunities. AI is increasingly being explored as a tool to support clinical decision-making and improve the quality of patient care. In obstetric sonography, for instance, it has demonstrated encouraging results in tasks such as image analysis and diagnostic interpretation [7]. ChatGPT also shows potential as a supportive tool in gynecologic oncology, providing responses that are not only accurate and comprehensive but also empathetic. These qualities may help both clinicians and patients better understand and manage care [8].

Pregnancy often involves natural feelings of anxiety and concern for expectant mothers [9]. During this time, ChatGPT can serve as a helpful tool by providing information and accurate and reliable answers to common obstetric questions in approximately 80% of cases, demonstrating its potential as a helpful tool in obstetrics [10].

One of the most important complications of pregnancy is gestational diabetes. Gestational diabetes affects about 14% of pregnancies worldwide; its incidence varies depending on risk factors and screening and diagnosis methods, and it is rising in tandem with type 2 diabetes and obesity [11,12]. A ChatGPT integrated into a national digital health platform answered 88.51% of gestational diabetes mellitus (GDM) questions successfully and seems to be a suitable solution for retrieving useful information to quickly answer patients' questions about GDM [13].

This study aimed to evaluate the responses of three different AI models, including ChatGPT (GPT-3.5 (OpenAI, San Francisco, USA), GPT-4o (OpenAI, San Francisco, USA)) and DeepSeek V3 0324 (DeepSeek AI, Beijing, China), to 24 critical questions related to GDM. Two perinatologists assessed the answers provided by these AI models for accuracy, completeness, and clinical reliability. These AI-generated responses were then compared with those given by obstetrics and gynecology (OB/GYN) residents to explore the potential role of these AI models in managing GDM. Comparing AI models with residents is particularly relevant, as residents often represent the first line of clinical communication with patients. They provide a realistic benchmark against which AI’s potential role in medical education and patient care can be evaluated.

## Materials and methods

This is a comparative cross-sectional study designed to evaluate and compare the accuracy and completeness of responses provided by three AI chatbots and OB/GYN residents to common patient questions regarding gestational diabetes in pregnancy.

A total of 24 common questions from patients with gestational diabetes who visited the Perinatology Clinic affiliated with Shiraz University of Medical Sciences between April 2024 and August 2024 were collected. A standard questionnaire was developed in collaboration with a statistician, a computer science specialist, and two obstetrics and gynecology faculty experts. The Perinatology Clinic is the only active subspecialty diabetes perinatology center in Shiraz, with approximately 60 pregnant women with gestational diabetes attending weekly; the selected questions reflect common concerns encountered in routine practice. The 24 questions were derived from the American College of Obstetricians and Gynecologists (ACOG) FAQ on gestational diabetes (https://www.acog.org/womens-health/faqs/gestational-diabetes) and were reviewed and adapted by two senior perinatologists to reflect the questions commonly raised by patients in our clinic.

Eight third-year OB/GYN residents were recruited as a convenience sample from those present at the Perinatology Clinic of Shiraz University of Medical Sciences during the study period (September-November 2024). Inclusion criteria included active participation in clinical duties during this time, while residents on leave were excluded. The residents were instructed to complete the questionnaire but were not informed of the study aim (comparison with AI models), in order to minimize potential bias.

These patient questions were collected prospectively from women with gestational diabetes who visited the clinic during the study period. They were selected consecutively to minimize selection bias.

The responses from the two study groups were collected and provided to two faculty members from the Department of Obstetrics and Gynecology, each with more than 10 years of experience. These two perinatologists independently reviewed the responses given by the residents and the AI models. The responses were rated for accuracy and completeness on a 5-point Likert scale (1: completely disagree, 2: partially disagree, 3: neutral, 4: partially agree, 5: completely agree). To evaluate accuracy and completeness, they used the American College of Obstetricians and Gynecologists' Guidelines for the Diagnosis and Treatment of Gestational Diabetes, standard perinatology reference books, and their years of medical experience. Importantly, the two perinatologists were blinded to the source of the responses (resident vs. AI) to avoid assessment bias. In cases of disagreement, differences were resolved through discussion to ensure consistent scoring. The AI models (GPT-3.5, GPT-4o, and DeepSeek V3 0324) were accessed between May and August 2024. Each model was given the same standardized prompt for each question: "Please provide a concise, evidence-based response suitable for a pregnant woman with gestational diabetes, based on the latest ACOG guidelines." Other contemporary models (e.g., Claude, MedPaLM) were not included because they were not publicly accessible or lacked stable API access at the time of data collection.

This study was conducted in accordance with the ethical principles of the Declaration of Helsinki. The research protocol was reviewed and approved by the Institutional Review Board (IRB) of Shiraz University of Medical Sciences (Approval Code: IR.SUMS.REC.1403.197, Approval Date: August 2024). The requirement for informed consent was waived by the IRB due to the use of de-identified data. Confidentiality and anonymity of participant data were strictly maintained.

Statistical analysis

All data were analyzed using IBM SPSS Statistics for Windows, Version 29 (Released 2024; IBM Corp., Armonk, New York, United States). Descriptive statistics (means, standard deviations, and 95% confidence intervals) were calculated for accuracy and completeness scores. Independent-samples t-tests were performed to compare mean scores between residents and each AI model. A two-tailed significance level of p < 0.05 was considered statistically significant. In addition to p-values, effect sizes (Cohen’s d) were calculated to quantify the magnitude of differences. Inter-rater discrepancies between the two perinatologists were resolved through discussion to reach consensus. Inter-rater agreement was assessed qualitatively prior to consensus; formal kappa statistics were not calculated due to the limited sample size, which is acknowledged as a limitation. The 24 standardized questions used in the study are presented in Appendix A.

## Results

Responses to 24 questions related to GDM were obtained from eight OB/GYN residents and three AI chatbots (GPT-3.5, GPT-4o, and DeepSeek V3 0324). Two independent faculty members assessed each response for accuracy and completeness on a 5-point Likert scale.

DeepSeek demonstrated the highest accuracy, with a mean score of 4.81 (95% CI, 4.69-4.93). GPT-4o followed with 4.69 (95% CI, 4.54-4.84), GPT-3.5 scored 4.67 (95% CI, 4.49-4.85), while residents scored significantly lower at 3.64 (95% CI, 3.33-3.95). Independent-samples t-tests confirmed that all AI models outperformed residents in accuracy: GPT-3.5 (t(46) = 5.94, p < 0.001), GPT-4o (t(46) = 6.31, p < 0.001), and DeepSeek (t(46) = 7.28, p < 0.001) (Table 1).

**Table 1:** Comparison of mean accuracy and completeness scores ns: not significant; ***: p < 0.001

Model	Mean Accuracy Score	Mean Completeness Score	p-Value (Completeness vs. Residents)
Residents	3.64	2.05	Reference
GPT-3.5	4.67	2.83	0.058 (ns)
GPT-4o	4.69	4.00	< 0.001 (***)
DeepSeek V3 0324	4.81	4.75	< 0.001 (***)

For completeness, DeepSeek again achieved the highest score (4.75; 95% CI, 4.66-4.84), followed by GPT-4o (4.00; 95% CI, 3.81-4.19) and GPT-3.5 (2.83; 95% CI, 2.64-3.02). Residents scored the lowest (2.05; 95% CI, 1.77-2.33). Statistical testing showed that GPT-4o (t(46) = 11.92, p < 0.001) and DeepSeek (t(46) = 18.99, p < 0.001) were significantly more complete than residents, while the difference between GPT-3.5 and residents did not reach statistical significance (t(46) = 1.95, p = 0.058).

Residents frequently omitted critical details, such as postpartum screening timing (68% of responses) and neonatal hypoglycemia risks (41% of responses). GPT-3.5 provided outdated guidelines in 22% of responses, while DeepSeek and GPT-4o consistently provided up-to-date information. For complex questions (e.g., insulin titration), DeepSeek maintained high accuracy (4.62; 95% CI, 4.48-4.76), whereas resident performance declined (2.89; 95% CI, 2.56-3.22).

**Figure 1:**
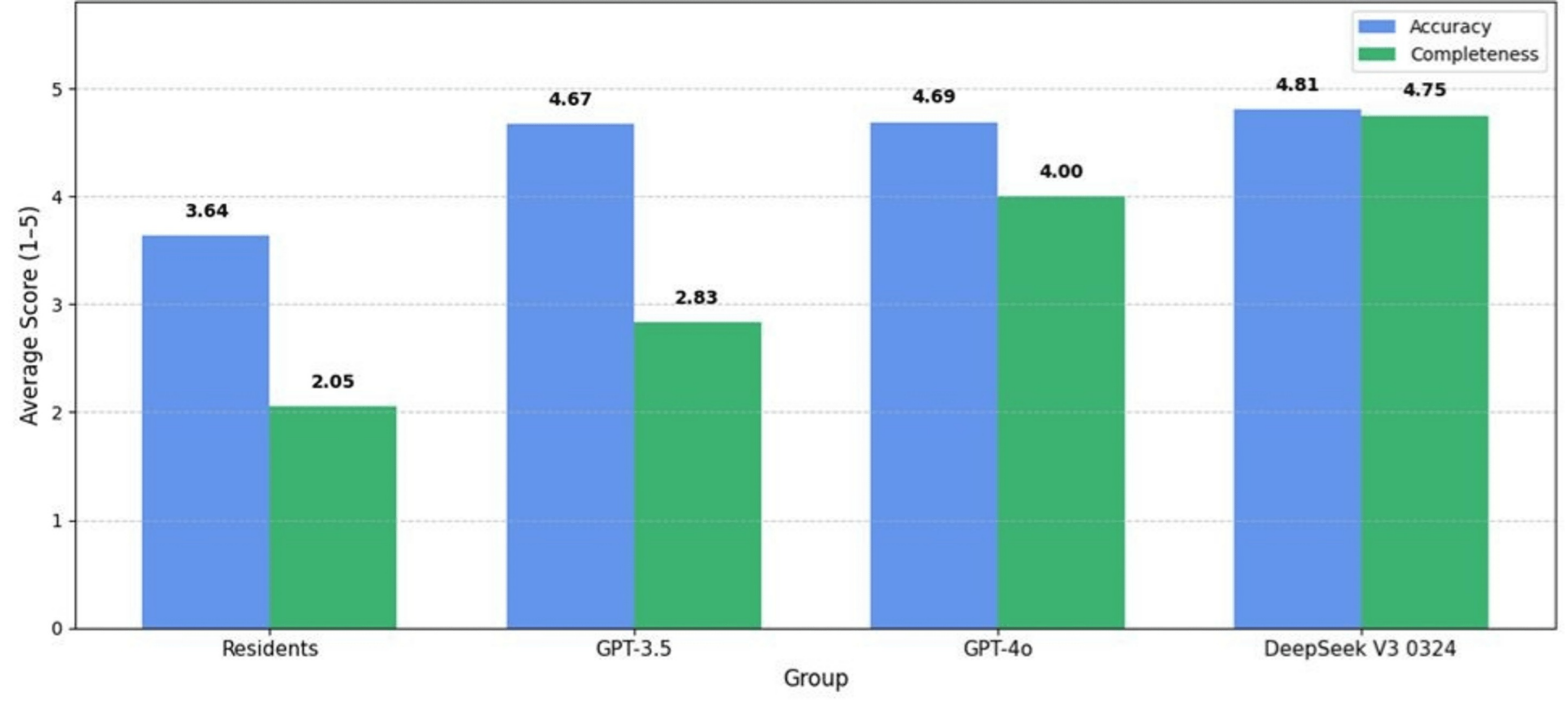
Comparison of mean accuracy and completeness scores among residents and AI models All AI models showed significantly higher accuracy compared to residents (p < 0.001). Completeness was significantly greater for GPT-4o and DeepSeek V3 0324 (p < 0.001). AI: Artificial intelligence

**Figure 2:**
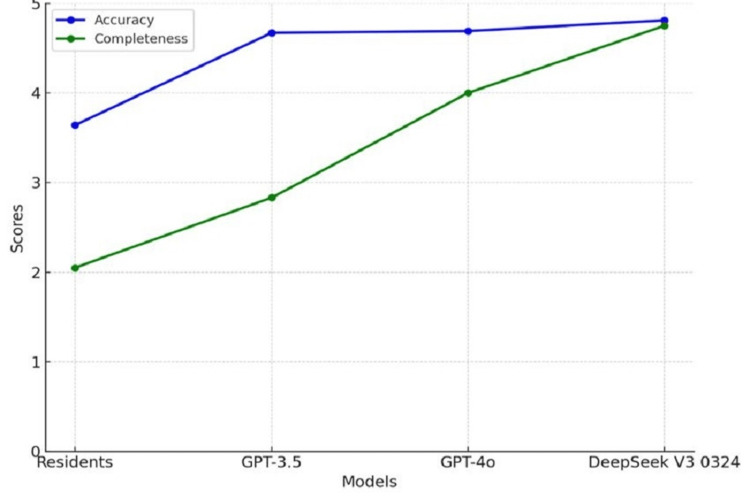
Trends in accuracy and completeness scores across evaluated models Notable improvement in both metrics is observed with newer AI models AI: Artificial intelligence

Among the AI models, DeepSeek V3 0324 achieved the highest scores in both accuracy and completeness, with minimal variability across the 24 questions. A graphical comparison (Figures 1, 2) illustrates these differences between resident and AI model performance.

Beyond p-values, effect sizes indicated practically meaningful differences: for accuracy, Cohen’s d was 1.72 (GPT-3.5 vs residents), 1.82 (GPT-4o vs residents), and 2.10 (DeepSeek vs residents); for completeness, d was 1.38, 3.44, and 5.48, respectively.

## Discussion

GDM is among the most common complications during pregnancy, affecting approximately one in six pregnancies worldwide [12]. Effective prenatal care, along with strong support for both the expectant mother and her family, as well as individualized nursing guidance, plays a vital role in minimizing health risks associated with GDM [14]. Naturally, receiving a diagnosis like GDM often brings a flood of questions from expectant mothers, ranging from how to manage diet and medications to understanding potential risks for their baby. While these concerns have traditionally been addressed by physicians, AI, particularly in the form of chatbots and predictive technologies, is increasingly stepping in to provide additional support. Emerging evidence suggests that chatbots can help answer patient questions. In particular, Reynolds and colleagues have underscored the potential of ChatGPT in assisting healthcare providers with the creation of patient education materials. Such resources may improve patient comprehension, encourage adherence to treatment plans, and contribute to reducing physician workload and burnout [15].

Huang et al. highlight the potential of ChatGPT in providing accurate diabetes information, but stress the need for further research, quality oversight, and real-time updates to ensure reliability and relevance [16].

AI is also getting better at talking to patients. In a notable comparison of responses to health-related questions, AI-generated answers were rated more helpful and empathetic than those from physicians. In fact, 78.6% of the AI responses were rated “good” or “very good,” compared to only 22.1% for doctors. Even more surprising was that AI was perceived as more empathetic; 45% of its responses were rated as such, versus just 4.6% for physicians [4].

Recent studies show that chatbots can offer real benefits to pregnant women. They provide trustworthy health information, help support mental well-being, increase understanding of prenatal care, and often improve patient satisfaction [17-21]. However, the reliability of these benefits depends greatly on the continuous updating of AI systems and the careful validation of their medical content.

 AI has also made notable progress in identifying GDM early on. By analyzing common clinical details, like age, body mass index (BMI), and blood glucose levels, some machine learning models can flag women at risk with impressive accuracy [22]. This is particularly valuable in places where specialist care might not be readily accessible. Still, despite these advances, no research has yet explored how chatbots respond to questions from pregnant women living with GDM.

Our study showed that chatbots can provide accurate and complete answers to many questions asked by pregnant patients with gestational diabetes. DeepSeek and GPT-4o, in particular, showed significantly higher completeness scores, suggesting that newer models may better synthesize clinical guidelines into patient-friendly language. These answers will become more complete and accurate as these chatbots are updated. In terms of accuracy, they may not be much different from the answers given by doctors, but they generally showed a higher level of completeness, although this must be interpreted cautiously given the study’s limitations. It is also possible that the observed differences partly reflect unequal baseline knowledge; AI models draw from vast databases, while residents rely on limited clinical experience within specific rotations. Beyond their performance metrics, these findings also carry implications for medical education. AI chatbots may serve as supplementary tools for resident training, helping them practice patient communication and clinical reasoning. Nevertheless, they should be used as educational aids rather than decision-making authorities.

From an ethical and medico-legal standpoint, any integration of AI tools into clinical communication must ensure clinician oversight, data transparency, and accountability for potential misinformation. Healthcare providers remain responsible for verifying AI-generated content before sharing it with patients. Thus, AI should be integrated carefully, complementing but not replacing traditional clinical training.

Limitations

This study has several limitations. First, the sample size was small and limited to a single center, which may affect the generalizability of findings. Second, although the perinatologists were blinded, other forms of bias may remain, such as residents being aware of evaluation. Third, only accuracy and completeness were assessed, while dimensions such as empathy or clarity of communication were not measured. Fourth, inter-rater reliability was qualitatively assessed but not quantified due to the small sample size, which may affect reproducibility. Future studies with larger, multicenter samples and additional outcome measures are warranted. Moreover, this study did not assess patient-centered outcomes such as satisfaction, trust, or health behaviors following AI-guided information, which represent important areas for future research. Additionally, differences in informational scope between AI models and human trainees may have influenced results; further research should address this methodological limitation.

## Conclusions

In this single-center study using 24 standardized patient questions on GDM, AI chatbots, particularly DeepSeek and GPT-4o, demonstrated higher mean scores for accuracy and completeness than OB/GYN residents. These differences were statistically significant and accompanied by substantial effect sizes, suggesting practical as well as statistical importance. However, given the study’s single-center design, small resident sample, and differences in informational scope between model training data and resident clinical experience, these results should be considered preliminary. AI systems therefore appear most appropriate as supervised adjuncts to support patient education and resident training rather than as replacements for clinicians; their outputs should be routinely validated and interpreted within a clinical context. Future multicenter studies with larger samples and patient-centered outcomes are needed to confirm these findings and to define safe, practical pathways for implementation.

## Appendices

Appendix A

What is gestational diabetes?

What causes GD?

If I develop GD, will I always have diabetes?

What are the risk factors for GD?

Who is most often affected?

How can GD affect a pregnant woman?

What other conditions can a woman with GD develop?

How can GD affect a baby?

Will I be tested for GD?

If I have GD during pregnancy, how will I manage it?

How do I track blood sugar levels?

Should I change my diet if I have GD?

Will regular exercise help me control GD?

Will I need to take medication to control my GD?

Will I need tests to check the health of my fetus?

Will GD affect the delivery of my baby?

What are the future health concerns for women who have had GD?

What are the future health concerns for children?

If I have GD, is there anything I should do after my pregnancy?

Can I use insulin?  

What are the risks of insulin to the fetus?

Can I use oral medications instead of insulin?

What can I do to control my blood sugar besides following a diet?

When is the termination of pregnancy for a pregnant woman with GDM?

## References

[1] Brocklehurst P (2016). A study of an intelligent system to support decision making in the management of labour using the cardiotocograph - the INFANT study protocol. BMC Pregnancy Childbirth.

[2] Ray PP (2023). ChatGPT: A comprehensive review on background, applications, key challenges, bias, ethics, limitations and future scope. Internet Things Cyber-Phys Syst.

[3] Barrington NM, Gupta N, Musmar B (2023). A bibliometric analysis of the rise of ChatGPT in medical research. Med Sci (Basel).

[4] Ayers JW, Poliak A, Dredze M (2023). Comparing physician and artificial intelligence chatbot responses to patient questions posted to a public social media forum. JAMA Intern Med.

[5] Tan S, Xin X, Wu D (2024). ChatGPT in medicine: prospects and challenges: a review article. Int J Surg.

[6] Goodman RS, Patrinely JR, Stone CA Jr (2023). Accuracy and reliability of chatbot responses to physician questions. JAMA Netw Open.

[7] Kim HY, Cho GJ, Kwon HS (2023). Applications of artificial intelligence in obstetrics. Ultrasonography.

[8] Reicher L, Lutsker G, Michaan N, Grisaru D, Laskov I (2025). Exploring the role of artificial intelligence, large language models: comparing patient-focused information and clinical decision support capabilities to the gynecologic oncology guidelines. Int J Gynaecol Obstet.

[9] Domínguez-Solís E, Lima-Serrano M, Lima-Rodríguez JS (2021). Non-pharmacological interventions to reduce anxiety in pregnancy, labour and postpartum: a systematic review. Midwifery.

[10] Peled T, Sela HY, Weiss A, Grisaru-Granovsky S, Agrawal S, Rottenstreich M (2024). Evaluating the validity of ChatGPT responses on common obstetric issues: potential clinical applications and implications. Int J Gynaecol Obstet.

[11] Sweeting A, Hannah W, Backman H (2024). Epidemiology and management of gestational diabetes. Lancet.

[12] Sweeting A, Wong J, Murphy HR, Ross GP (2022). A clinical update on gestational diabetes mellitus. Endocr Rev.

[13] Sagstad MH, Morken NH, Lund A, Dingsør LJ, Nilsen AB, Sorbye LM (2022). Quantitative user data from a chatbot developed for women with gestational diabetes mellitus: observational study. JMIR Form Res.

[14] Topkara S, Soysal Ç (2024). The effect of diabetes education on maternal and neonatal outcomes in pregnant women diagnosed with gestational diabetes. BMC Pregnancy Childbirth.

[15] Reynolds K, Tejasvi T (2024). Potential use of ChatGPT in responding to patient questions and creating patient resources. JMIR Dermatol.

[16] Huang C, Chen L, Huang H (2023). Evaluate the accuracy of ChatGPT's responses to diabetes questions and misconceptions. J Transl Med.

[17] Su B, Jones R, Chen K (2025). Chatbot for patient education for prenatal aneuploidy testing: a multicenter randomized controlled trial. Patient Educ Couns.

[18] McAlister K, Baez L, Huberty J, Kerppola M (2025). Chatbot to support the mental health needs of pregnant and postpartum women (moment for parents): design and pilot study. JMIR Form Res.

[19] Nguyen QC, Aparicio EM, Jasczynski M (2024). Rosie, a health education question-and-answer chatbot for new mothers: randomized pilot study. JMIR Form Res.

[20] Chung K, Cho HY, Park JY (2021). A chatbot for perinatal women's and partners' obstetric and mental health care: development and usability evaluation study. JMIR Med Inform.

[21] Mancinelli E, Magnolini S, Gabrielli S, Salcuni S (2024). A chatbot (Juno) prototype to deploy a behavioral activation intervention to pregnant women: qualitative evaluation using a multiple case study. JMIR Form Res.

[22] Zhang Z, Yang L, Han W (2022). Machine learning prediction models for gestational diabetes mellitus: meta-analysis. J Med Internet Res.

